# Supplementary figures and images for: Longitudinal examination of perfusion and angiogenesis markers in primary colorectal tumors shows distinct signatures for metronomic and maximum-tolerated dose strategies
Source: Neoplasia. 2022 Jul 25;32:100825. doi: 10.1016/j.neo.2022.100825 (PMC9326335; doi:10.1016/j.neo.2022.100825)

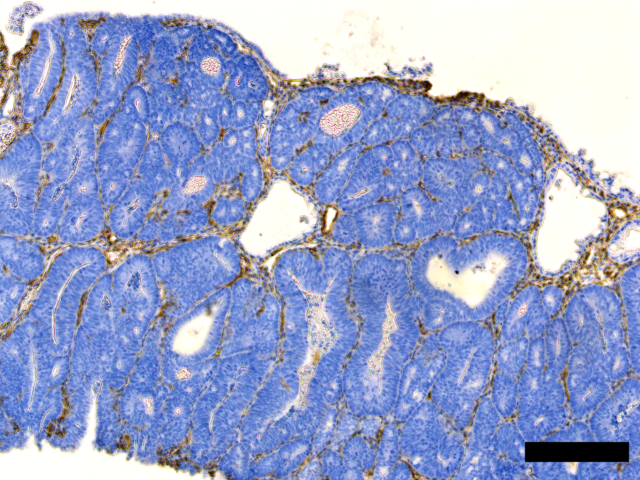

Supplement: Supplementary Data S1 — Supplementary Raw Research Data. This is open data under the CC BY license http://creativecommons.org/licenses/by/4.0/ [file mmc1.zip › Primary_tumor_longitudinal_chemotherapy-main/figures/CG_Nestin-scaled.png]

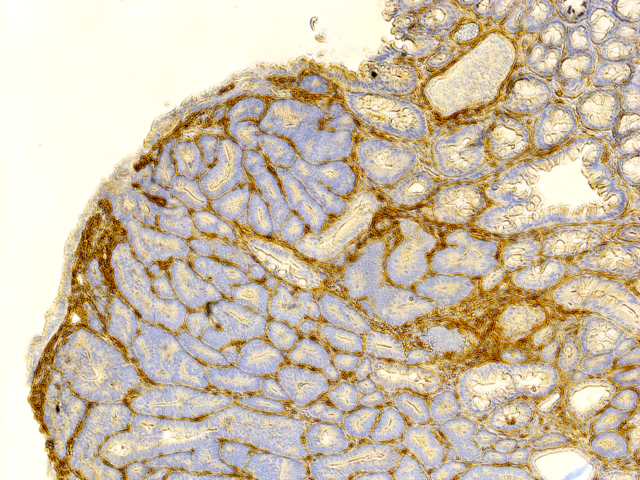

Supplement: Supplementary Data S1 — Supplementary Raw Research Data. This is open data under the CC BY license http://creativecommons.org/licenses/by/4.0/ [file mmc1.zip › Primary_tumor_longitudinal_chemotherapy-main/figures/MET_Nestin-scaled.png]

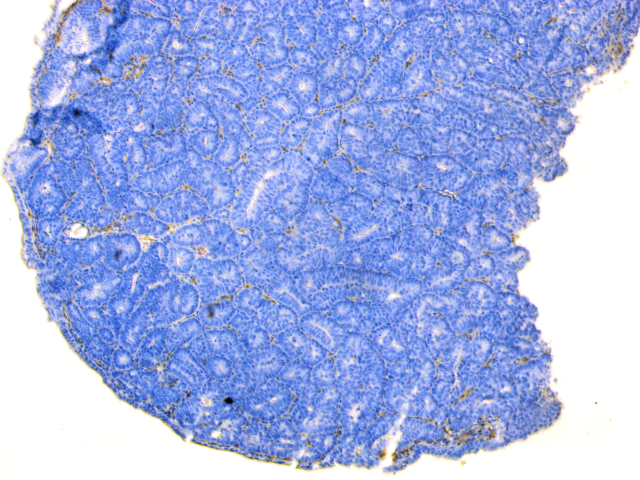

Supplement: Supplementary Data S1 — Supplementary Raw Research Data. This is open data under the CC BY license http://creativecommons.org/licenses/by/4.0/ [file mmc1.zip › Primary_tumor_longitudinal_chemotherapy-main/figures/MTD_Nestin-scaled.png]

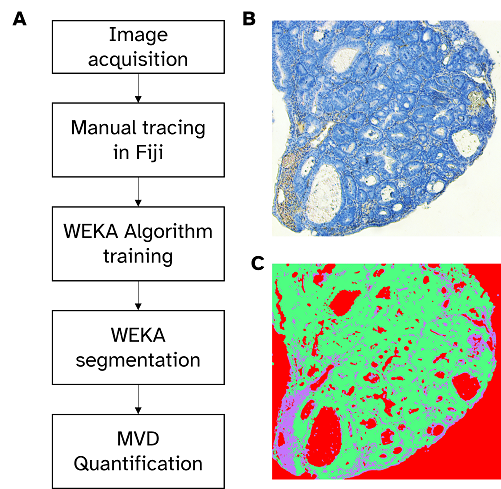

Supplement: Supplementary Data S1 — Supplementary Raw Research Data. This is open data under the CC BY license http://creativecommons.org/licenses/by/4.0/ [file mmc1.zip › Primary_tumor_longitudinal_chemotherapy-main/figures/MVD_quant_Appendix.png]

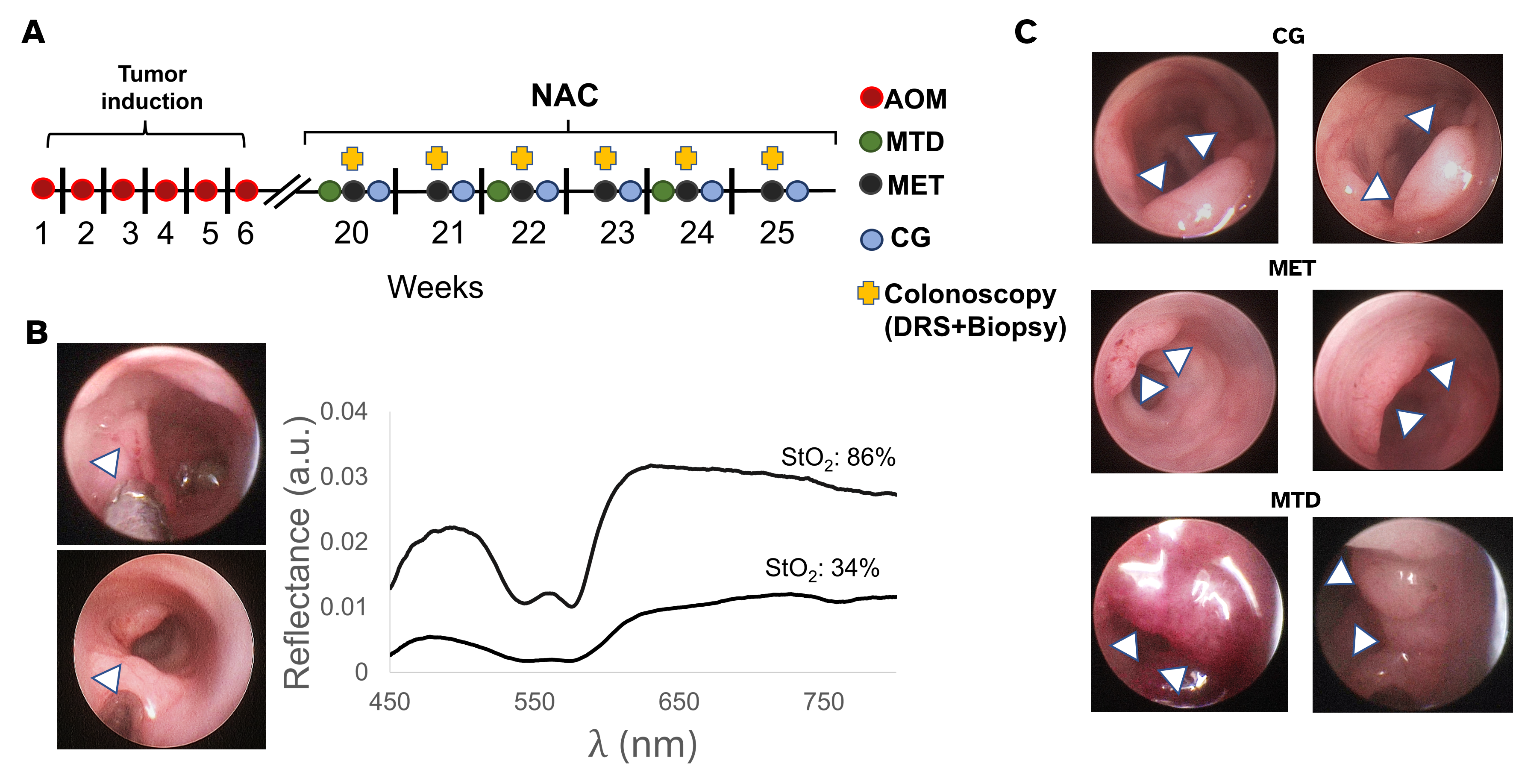

Supplement: Supplementary Data S1 — Supplementary Raw Research Data. This is open data under the CC BY license http://creativecommons.org/licenses/by/4.0/ [file mmc1.zip › Primary_tumor_longitudinal_chemotherapy-main/figures/study_design.tif]

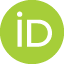

Supplement: Supplementary Data S1 — Supplementary Raw Research Data. This is open data under the CC BY license http://creativecommons.org/licenses/by/4.0/ [file mmc1.zip › Primary_tumor_longitudinal_chemotherapy-main/manuscript/latex_docs/orcid.png]
